# Subcutaneous Immunoglobulin for Antibody Deficiency in Antineutrophil Cytoplasmic Antibody (ANCA)-associated Vasculitis

**DOI:** 10.7759/cureus.6367

**Published:** 2019-12-12

**Authors:** Sam Kant, Antoine Azar, Eric J Gapud, Brendan Antiochos, Rebecca Manno, Philip Seo, Duvuru Geetha

**Affiliations:** 1 Nephrology, Johns Hopkins Hospital, Johns Hopkins University School of Medicine, Baltimore, USA; 2 Allergy and Immunology, Johns Hopkins Hospital, Johns Hopkins University School of Medicine, Baltimore, USA; 3 Rheumatology, Johns Hopkins Hospital, Johns Hopkins University School of Medicine, Baltimore, USA; 4 Medicine, Johns Hopkins Hospital, Johns Hopkins University School of Medicine, Baltimore, USA

**Keywords:** immunoglobulin, antibody deficiency, antineutrophil cytoplasmic antibody (anca) vasculitis

## Abstract

Objectives

Intravenous immunoglobulin G (IVIG) is used to treat antineutrophil cytoplasmic antibody (ANCA) patients with recurrent infections as a result of hypogammaglobulinemia (HG) induced by treatment regimens. We sought to characterize clinical features, treatment, and outcomes for patients treated with the novel subcutaneous IgG (SCIG) for the aforementioned purpose.

Methods

We conducted a retrospective study of 136 patients in our ANCA database to identify patients with recurrent infections and HG subsequently treated with SCIG. Patient demographics, serologies, treatment, and immunological parameters were assessed.

Results

Of 136 patients, four were treated with SCIG. All were Caucasian, proteinase-3 (PR3)-positive, and the majority (n = 3) were females. All patients had pulmonary involvement, and regimens of cyclophosphamide (CYC) and/or rituximab (RTX) were employed for induction and remission. Three patients each experienced recurrent bouts of respiratory tract infections and shingles. Ig levels (G, M, and A) were reduced in all patients, except for one patient who had normal IgA levels. CD19/20 cells were depleted and CD3/4/8/NK cells were preserved in all patients. Three patients had no discernible antibody response to the pneumococcal vaccine (specific pneumococcal serotypes measured pre- and post-vaccine). The mean duration elapsed between the first rituximab administration and commencement of SCIG was 7.2 years. The IgG level normalized and none of the patients had a recurrence of infection since the initiation of SCIG.

Conclusion

This data, albeit preliminary, is the first series that demonstrates SCIG can be a reliable alternative to IVIG in ANCA patients with recurrent infections secondary to HG. Early identification of this subset of patients is likely to mitigate infectious risks, associated morbidity, and hospitalization.

## Introduction

Antineutrophil cytoplasmic antibody (ANCA)-associated vasculitis (AAV) is frequently treated with regimens that include cyclophosphamide (CYC) and/or rituximab (RTX). Both therapies have been implicated in the development of hypogammaglobulinemia (HG) [1-3]. 

RTX is a chimeric monoclonal antibody directed against the CD20 antigen on B cells. It is associated with HG because it reduces plasma cell precursors [4]. Studies have shown that a significant proportion of patients develop HG when they receive repeated treatment with RTX for remission maintenance, independent of cumulative dose [2]. In two retrospective studies of remission maintenance with RTX, severe infections and HG were frequent adverse events: 26% to 29% had severe infections and 41% to 45% had HG [2, 5-6]. Severe HG has been associated with an increased risk for infection requiring hospitalization in patients with AAV [7]. The risk of severe infection seems to be driven primarily by the reduction in IgG associated with rituximab therapy [8]. 

Immunoglobulin replacement therapy (IRT) has been employed for the treatment of RTX-induced HG [9-10], and its use leads to a reduction in infectious events and the need for antibiotics [11]. When IRT is initiated, intravenous immunoglobulin (IVIG) is the formulation that has been used uniformly in all case series. However, IVIG therapy is associated with systemic adverse effects, including infusion reaction, thromboembolism, acute kidney injury, and osmotic nephrosis. The renal side effects have been linked to certain IVIG formulations stabilized with sucrose [3]. Subcutaneous immunoglobulin (SCIG) does not contain sucrose and has been proposed as an alternative to IVIG. SCIG has been used to treat primary immune deficiency diseases, and one formulation, Hizentra® (CSL Behring AG, King of Prussia, PA, USA), has been FDA-approved for the treatment of chronic inflammatory polyneuropathy. SCIG has been used off-label to treat autoimmune diseases. The use of a subcutaneous formulation in AAV has not been explored. In addition, there is a lack of information on the optimal way to assess the risk of infection and guide IRT in AAV patients with HG and recurrent infections. We sought to characterize AAV patients treated with SCIG in our center, provide a framework for the assessment of antibody deficiency and the eventual institution of IRT, and report subsequent outcomes. 

This work was previously presented at the 19th International Vasculitis and ANCA Workshop, April 7-10, Philadelphia, PA (Abstract #315: Kant S, Azar A, Gapud E, Seo P, Geetha D: Use of Subcutaneous IgG to Treat Hypogammaglobinemia in ANCA-Associated Vasculitis).

## Materials and methods

We conducted a retrospective study of 136 AAV patients from our vasculitis center's institutional review board (IRB)-approved database to identify patients with recurrent infections and HG who were subsequently treated with SCIG. Patient demographics were recorded and included age, gender, and ethnicity, along with disease-specific variables, such as ANCA serology, organ involvement, and renal function (initial and from the last follow-up visit). 

Treatment regimens for induction and maintenance, along with details of exposure to CYC and RTX and time elapsed since last RTX administration and continuation post-RTX, were noted. A detailed infection history was obtained. Immunological parameters, including serum levels of IgG, IgA, and IgM and lymphocyte subsets (CD 19/20, CD3/4, CD3/CD8, CD16/CD56 NK cells), were assessed. IgG antibody response to pneumococcal polysaccharide vaccine, diphtheria, and tetanus prior to SCIG therapy were tested. The occurrence of infection and the use of RTX for the treatment of AAV post-initiation of SCIG were recorded.

## Results

Of the 136 patients, four were treated with SCIG. Two patients were less than 30 years old and two were more than 50 years of age. All four patients were Caucasian and were PR3-ANCA-positive; three of the patients were female. All patients had pulmonary involvement; three also had renal involvement. Two patients had received cyclophosphamide (CYC), one patient had received both CYC and RTX, and one received RTX alone for remission induction (Table 1). 

**Table 1:** Treatment Regimens for ANCA-associated Vasculitis, IgG Levels, and Number of Infections Prior to Commencement of SCIG ANCA: antineutrophil cytoplasmic antibody; AZA: azathioprine; CYC: cyclophosphamide; Ig: immunoglobulin; PLEX: plasmapheresis; RTX: rituximab; S: steroids; SCIG: subcutaneous immunoglobulin

Patient ID	Induction	Maintenance	RTX details	Duration of RTX cessation prior to SCIG (months)	Ig levels prior to SCIG (mg/dL) (Normal range 700 - 1,600 mg/dL)	Number of infections
1	S + CYC + PLEX + RTX	AZA	Induction 375 mg/m^2^ q week x 4	95	299	5
2	S + RTX	RTX	Seven courses of induction 375 mg/m^2^ q week x 4 for initial disease and six relapses. Maintenance q six months	Ongoing RTX use	484	2
3	S + CYC	RTX	Induction 375 mg/m^2^ q week x 4; one dose of 500 mg for recurrence	10	318	2
4	S + CYC	RTX	Maintenance 1,000 mg q six months	Ongoing RTX use	392	5

Rituximab was used for remission maintenance for three of these patients, while a single patient received azathioprine for remission maintenance. With regards to recurrent infections, three patients each experienced recurrent bouts of respiratory tract infections and shingles prior to the initiation of SCIG. Influenza, Pneumocystis jirovecii, and Candida pneumonia affected one patient each (Table 2). 

**Table 2:** Types of Recurrent Infections Encountered Prior to the Commencement of Subcutaneous IgG

Infection (Number of patients affected)
Pneumonia (bacterial and viral) (1)
Herpes zoster (shingles) (3)
Sinusitis/upper respiratory tract infections (bacterial and viral) (2)
Influenza (1)
Pneumocystis jirovecii pneumonia (1)
Candida pneumonia (1)
Candida - oral thrush (1)

Immunoglobulin levels (G, M, and A) were reduced in all patients, except for one patient who had a normal IgA level. CD19/20 cells were depleted and CD3/4/8/NK cells were preserved in all patients. All four patients had an impaired antibody response to the pneumococcal vaccine (specific pneumococcal serotypes measured pre- and post-vaccine). The median duration elapsed between the first RTX administration and the commencement of SCIG was 7.5 years (range: 5 to 9 years). The median follow-up post-SCIG was seven months (range: 3 to 60 months). SCIG was well tolerated without any adverse events. The use of SCIG allowed continued use of RTX for relapse prevention in three patients who were felt to be at high risk for relapse by the treating physicians. Of these three patients, Patient #2 in Table 1 (who has been on RTX for about 59 months and has had relapsing granulomatosis with polyangiitis (GPA)) has been relapse-free with less frequent dosing of RTX for remission maintenance. None of the patients had a recurrence of infection since the initiation of SCIG. 

## Discussion

In this report, we describe four patients with HG and antibody deficiency in the setting of RTX use for AAV and provide a framework for the evaluation of HG in these patients. This is the first series in the literature to describe AAV patients treated with SCIG. Induction and maintenance regimens for AAV have been the subject of numerous trials over the past decade. An inadvertent consequence of therapy, especially RTX, can be HG. RTX depletes pre-plasma B cells and this may lead to decreased repopulation of plasma cells, resulting in decreased production of immunoglobulins [7]. 

HG occurs in > 50% of AAV patients treated with RTX [12] but rarely occurs in patients with rheumatoid arthritis treated with the same drug [9]. This difference may be related to older age, concomitant use of other immunosuppressive medications, or uncharacterized B cell dysfunction in AAV. In 179 patients with AAV and systemic lupus erythematosus (SLE) treated with RTX (of which 69% had prior cyclophosphamide exposure and 58% were on concomitant immunosuppression), 23% developed de novo HG [4]. Low baseline immunoglobulin levels, prior cyclophosphamide exposure, and glucocorticoid therapy have been shown to be risk factors for RTX-induced HG [4, 8]. The majority of the patients in our study were treated with cyclophosphamide as induction and RTX as maintenance therapy, with two requiring repeat administrations of RTX for recurrence. 

Severe HG, in turn, has been shown to be associated with a higher risk of infections requiring hospitalization in patients with AAV [7]. In our cohort, levels of IgG, IgM, and IgA were decreased in all patients prior to initiation of SCIG, except for one patient with normal IgA levels. The majority of the patients had IgG levels of less than 400 mg/dL. 

Previous studies have shown a decline in IgG and IgM concentrations in AAV patients who received CYC followed by RTX [13]. In the same study, 21% of patients were started on Ig replacement because of severe bronchopulmonary infections and serum IgG concentrations < 500 mg/dL. This is in line with our findings where nearly all of the patients had recurrent upper and lower respiratory tract infections, along with herpes zoster. The latter infection was also seen in a significant proportion (23%) of AAV patients who had severe HG in another study [7].

There are currently no guidelines to direct IRT in AAV patients with HG. The Joint European League Against Rheumatism and European Renal Association-European Dialysis and Transplant Association (EULAR/ERA-EDTA) recommendations for the management of AAV recommend the involvement of an immunologist in the setting of persistent HG [14]. In the general setting, the severity of HG and functional antibody deficiency (an indicator of the impaired immune response) have been used to guide IRT. Half of the patients treated with IRT had functional antibody deficiencies in a cohort of RTX-treated patients [9]. We tested functional antibody deficiency by ascertaining baseline specific pneumococcal serotype antibodies and then measured the same six weeks post-administration of the 23-valent pneumococcal vaccine (PPV23). All of our patients had evidence of functional antibody deficiency in addition to HG. 

**Figure 1:**
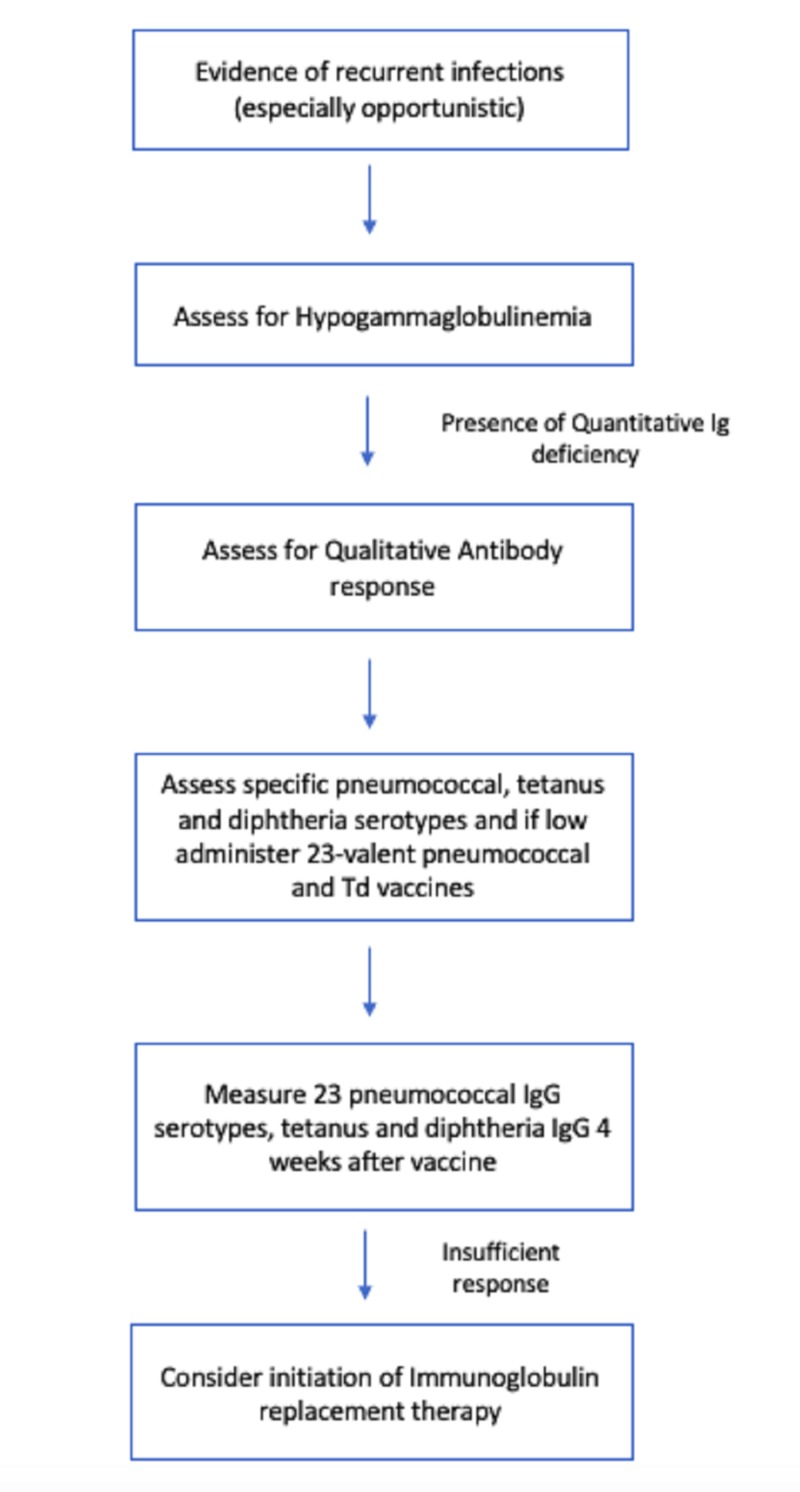
Proposed algorithm for treatment of hypogammaglobulinemia and recurrent infections in AAV patients AAV: antibody-associated vasculitis; SCIG: subcutaneous immunoglobulin; TD: tetanus-diphtheria

SCIG was then commenced in these patients with an excellent response as judged by no recurrence of infection in any patients (see Figure 1 for the algorithm for treatment). Furthermore, the use of SCIG allowed continued administration of RTX for relapse prevention in these patients. In a single patient in this cohort who had relapsing GPA with six disease flares, the use of SCIG was associated with a relapse-free disease state with less frequent dosing of RTX which may be due to the immunomodulatory effects of SCIG. SCIG has been shown to be safe, cost-effective, and greatly improves health-related quality of life (HRQL) [13, 15]. Although the aforementioned data has mostly been demonstrated in patients with primary antibody deficiency, our study is the first of its kind to show that SCIG is a reliable alternative to intravenous immunoglobulin (IVIG) in AAV patients with HG and recurrent infections. 

With respect to limitations, our study has a small subset of patients with short-term follow-up. Further studies are needed to assess long-term outcomes in larger patient cohorts. 

## Conclusions

In conclusion, it is imperative to consider IRT in AAV patients with hypogammaglobulinemia and recurrent infections post-therapy with RTX and/or CYC. SCIG appears to be safe in these patients and appears to reduce recurrent infections, which may, in turn, translate into enhanced outcomes for patients. 
